# Qatari Genotype May Contribute to Complications in Type 2 Diabetes

**DOI:** 10.1155/2020/6356973

**Published:** 2020-06-08

**Authors:** Youssra Dakroury, Stephen L. Atkin, Soha R. Dargham, Amal Robay, Juan Rodriguez-Flores, Ronald G. Crystal, Alexandra E. Butler

**Affiliations:** ^1^Weill Cornell Medicine-Qatar, PO Box 24144, Doha, Qatar; ^2^Royal College of Surgeons in Ireland, Bahrain; ^3^Department of Genetic Medicine, Weill Cornell Medicine, New York, USA; ^4^Diabetes Research Center (DRC), Qatar Biomedical Research Institute (QBRI), Hamad Bin Khalifa University (HBKU), Qatar Foundation (QF), Doha, Qatar

## Abstract

**Objective:**

There is increasing evidence of a strong genetic component in type 2 diabetes (T2DM) that may contribute to diabetes complications. Given the high prevalence of diabetes with its associated complications in the Middle East, we sought to determine if the genotype within a Middle East population may be contributory. Therefore, three genotype-based Qatari ancestral groups, Q1 Arab Bedouin, Q2 Asian/Persian, and Q3 sub-Saharan African, with a fourth admixed group were correlated with T2DM prevalence and its complications to determine if they differed between the 4 Qatari ancestries, particularly for the SLMAP allele-associated diabetic retinopathy.

**Methods:**

In this cross-sectional study, 398 Qatari subjects, 220 with and 178 without T2DM, were genotyped by Affymetrix 500k SNP arrays. Ancestry was correlated with diabetes complications.

**Results:**

398 subjects were included, the mean age was 49.8 years, and 56.8% were male. The genotype-based ancestry and T2DM prevalence were as follows: 164 (41.2%) with ancestry Q1, 60.4% with T2DM; 149 (37.4%) with ancestry Q2, 49.7% with T2DM; 31 (7.8%) with ancestry Q3, 61.3% with T2DM; and 54 (13.6%) with “admixed” ancestry, 51.9% with T2DM. For patients with diabetes, hypertension (*p* < 0.035) and retinopathy (*p* < 0.016) were greater in the Q3 ancestry.

**Conclusion:**

These data suggest that the genotype may contribute to complication risk, as exemplified by the increase in hypertension and retinopathy in the Q3 ancestry, though the SLMAP allele was not implicated; however, diabetes prevalence did not differ between the four Qatari ancestries.

## 1. Introduction

Type 2 diabetes (T2DM) is a complex disease with a heterogeneous genetic component that has not been fully determined, hindering attempts at personalized medicine [1]. Stratification of discrete diabetes subtypes has been shown to predict disease trajectory, for example, the identification of diabetic retinopathy in insulin-deficient patients [1], suggesting that differing genotypes within a population may be important in diabetes complication development and in disease progression. Diabetes is particularly prevalent in the Middle East with approximately 20% of the population having T2DM, a figure 2-3 times higher than the world average [2]. As a consequence, the burden of diabetes complications is increased, with 43% of dialysis patients having diabetic nephropathy [3].

Next-generation exome sequencing has identified three major genotype-based ancestry groups within the Qatari population (Q1 Bedouin, Q2 Persian-South Asian, and Q3 African) and has identified variants within genes that have effects on clinically significant Mendelian diseases [4–6]. Several of these Mendelian variants were segregated only in one Qatari ancestry group [5]. Studies have shown that the T2DM risk alleles in a European population were similar to those found in the Q1 and Q2 ancestries, with a specific SNP (rs4506565) being related to the admixed population, and it was concluded that the European T2DM SNPs did not contribute to the high prevalence of T2DM in Qatar [6, 7]. Others have looked specifically at the SLMAP gene in the Qatari population and found that the SLMAP C>T polymorphism is associated, as an independent risk factor for retinopathy [8].

Recent data has suggested that there are extensive differences in the frequency of pharmacogenetic variants in the Qatari population [9] leading to the hypothesis purported here that differing genotypes may affect differing frequencies of diabetes complications in this homogeneous population [10] and perhaps that the prevalence of diabetes within the genotypes may differ.

The aim of this study was to determine, in this Qatari population where there are distinct genotypes together with high prevalence of diabetes and its associated complications, whether the genotype may be a contributory factor in the development of diabetes complications.

## 2. Methods

### 2.1. Study Population

The recruitment strategy for the subjects in this cross-sectional study has been described before [6]. Briefly, all subjects were over the age of 30 and were minimum three-generation Qataris. Cases were excluded if any of the following were present: history of type 1 diabetes, maturity onset diabetes of the young (MODY), maternally inherited diabetes and deafness syndrome (MIDD), a first-degree relative with type 1 diabetes, or secondary diabetes [6]. The diagnosis of type 2 diabetes was made according to the WHO guidelines [11]; for inclusion in the type 2 diabetes cohort, at least one of the following was required: fasting plasma glucose > 7 mmol/l, HbA1c > 6.5%, or a diagnostic glucose tolerance test. Inclusion in the nondiabetic control group required a normal glucose tolerance test. 398 subjects who satisfied the Qatari ancestry criteria and with unambiguous assignment to a Qatari subpopulation were selected from a group visiting the health clinics at Hamad Hospital, Doha, Qatar, for a routine diabetes screening [5]. Nondiabetic subjects were comprised of relatives accompanying the type 2 diabetic (T2DM) subjects.

Exclusion criteria were a diagnosis of type 1 diabetes, gestational diabetes, or diabetes secondary to steroid treatment.

Three genotype-based Qatari ancestral groups were identified: Q1 Arab Bedouin, Q2 Asian/Persian, and Q3 sub-Saharan African, plus a fourth admixed group.

The study was approved by Weill Cornell IRB (IRB# 13-00063), and all participants provided written informed consent. The conduct of the trial was in accordance with ICH GCP and the Declaration of Helsinki.

### 2.2. Study Design

Following an overnight fast, blood samples were collected and weight and blood pressure were measured at the baseline visit. The fasting venous blood was collected into fluoride oxalate and serum gel tubes. Samples were separated by centrifugation at 2000g for 15 minutes at 4°C, and the aliquots were stored at -80°C within 1 hour of collection. Blood pressure was measured using an automated device (NPB-3900; Nellcor Puritan Bennett, Pleasanton, CA) during each study visit. Blood pressure measurements were performed after the subjects had been seated quietly for at least 5 minutes and with the right arm supported at the heart level. Three readings were taken, each at least 2 minutes apart, and then, the average of the readings was obtained. Dyslipidemia was defined as total cholesterol greater than 190 mg/dl (>4.9 mmol/l) and/or fasting triglycerides > 150 mg/dl (>1.7 mmol/l) untreated or if subjects were under treatment.

#### 2.2.1. Microvascular Disease

Diabetic retinopathy was diagnosed by fundoscopy. Diabetic neuropathy (DN) was diagnosed based on the vibration perception threshold (Neurothesiometer NU-1, Horwell, UK) of the great toe being >25 V [12].

Fasting plasma glucose (FPG) was measured using a Synchron LX 20 analyzer (Beckman-Coulter) according to the manufacturer's recommended protocol. HbA1c was measured on a COBAS analyzer according to the manufacturer's recommended protocol (Roche Diagnostics).

### 2.3. Qatari Genetic Subpopulation Genotyping

DNA was extracted from blood using the QIAamp DNA Blood Maxi Kit (Qiagen Sciences Inc., Germantown, MD). The 398 subjects were classified into the three genotype-based ancestries described in the Qatari population [4, 8, 9] using a TaqMan SNP Genotyping Assay (Life Technologies, Carlsbad, CA) for a previously described panel of 48 ancestry informative SNPs [4, 5, 8, 9]. The average genotype call rate was 96% and was analyzed in STRUCTURE with *K* = 3. The Q1, Q2, or Q3 population was assigned if the highest proportion was >65%; otherwise, subjects were classed as “admixed.” The SLMAP allele was noted specifically given its association with retinopathy in the Qatari diabetic population.

### 2.4. Study Outcomes

#### 2.4.1. Statistical Analyses

In a previous study, it was noted that genetic variation among the Qatari population was remarkably structured and that the Qatari can be largely divided into three primary affinity groups based on a studied population of 168 individuals [4]. As there are no studies on the Qatari genotype and diabetes complications to allow a formal power calculation, it was empirically determined that the sample size for both the diabetes and control populations needed to be not less than 168 individuals, and in this case, 220 were type 2 diabetes patients and 178 controls. Data trends were visually and statistically evaluated for normality. Nonparametric tests (Mann-Whitney *U* and Kruskal-Wallis tests) were applied on the data that violated the assumptions of normality when tested using the Kolmogorov-Smirnov test. ANOVA was undertaken with post hoc analysis. The Bonferroni correction was applied to account for multiple testing. Simple logistic regression was conducted to assess the risk factors for diabetic retinopathy and diabetic neuropathy. Odds ratios (OR) and their confidence intervals (CI) were reported. Statistical analysis was performed using SPSS for Windows, version 24.0. All values are given as mean ± SD or as mean with 95% CI unless otherwise specified.

## 3. Results

### 3.1. Baseline Characteristics

A total of 398 subjects were genotyped (Table 1). Of the 398 subjects (mean age 49.8 ± 10.6 years; 226 males and 172 females), 220 had type 2 diabetes and 178 did not. The BMI (mean 32.4 ± 4.0 versus 30.1 ± 3.8, *p* < 0.001, respectively) and HbA1c (median (interquartile range) 7.9 (11.2) versus 5.6 (4.6), *p* < 0.001, respectively) were both higher in those with diabetes than in controls.

The baseline demographic data for the cohort categorized according to genotype-based ancestry showed that they did not differ in age, BMI, HbA1c, glucose, or diabetes prevalence (Table 1).

### 3.2. Diabetes and Diabetes-Related Complications in relation to Qatari Genotype-Based Ancestry

In the 398 subjects, the mean age was 49.8 years. 226 (56.8%) subjects were male, and 172 (43.2%) were female. The distribution among ancestries was as follows: 164 (41.2%) with ancestry Q1, of whom 99 (60.4%) had T2DM; 149 (37.4%) with ancestry Q2, of whom 74 (49.7%) had T2DM; 31 (7.8%) with ancestry Q3, of whom 19 (61.3%) had T2DM; and 54 (13.6%) with “admixed” ancestry, of whom 28 (51.9%) had T2DM (Table 1). The prevalence of diabetes did not differ between the genotype-based ancestries (Table 1).

Hypertension was greater in the Q3 ancestry (71%) compared to Q1 (47%), Q2 (42.3%), or admixed (44.4%) (*p* < 0.035). Diabetic retinopathy was greater in the Q3 ancestry (35.5%) compared to Q1 (15.2%), Q2 (12.8%), or admixed (20.4%) (*p* < 0.016). No difference was found for dyslipidemia or neuropathy between the genotype-based ancestries (Table 2). Odds ratios for the risk factors for type 2 diabetes complications are shown in Table 3. The odds ratios indicate that both diabetic retinopathy and diabetic neuropathy are associated with age, HbA1c, fasting glucose, hypertension, and dyslipidemia but are not associated with gender or BMI.

Given the association of diabetic retinopathy with the Q3 ancestry found here and the previously reported role of SLMAP genetic variants in the susceptibility to diabetic retinopathy in the Qatari population, we questioned whether the Q3 ancestry might harbor an altered allele frequency that could explain this association. We therefore determined the allele frequency in the Q1, Q2, and Q3 ancestries but found no differences in the allele frequency between these ancestral groups (Table 4).

## 4. Discussion

Stratification of diabetes into multiple subtypes has shown that diabetes complications differed across them [13] and that there was a clustering of genetic associations. In accordance with these observations, it can be seen that both retinopathy and hypertension appeared to be linked to the Q3 sub-Saharan ancestry when compared with the Q1 Arab/Bedouin, Q2 Persian/South Asian, and admixed ancestries. Whilst this finding needs to be confirmed in a larger population, it can be seen that the genetic risk score for retinopathy based on the Q3 ancestry may be a potential step in developing personalized medicine in this population to screen for and prevent an increased risk of diabetic retinopathy. Diabetes did not associate with any specific genotype-based ancestry, in accordance with the T2DM risk allele studies already undertaken [6].

A different genetic profile to that of a Caucasian population for obesity has been reported in this Middle Eastern population [14]; however, there were no differences in BMI between the differing ancestries studied here. It is of interest that an association of the PPAR*γ*2 gene variant pro12Ala polymorphism with hypertension has been identified in this population, though there was no association with the Ala allele and obesity [15]; again, it is not known how this relates to the Q3 ancestry.

The prevalence of diabetes in Africa is reported to be 3.3%, with the sub-Saharan countries reported to be less than 5%, but there are 49 diverse sub-Saharan countries and it is recognized that these countries have some of the highest rates of undiagnosed diabetes [16]. By way of comparison, the regional prevalence in Europe is 8.8% (7.0-12.0%) whilst that of the Middle East is 9.6% (6.7-12.7%) [16]. In terms of diabetic retinopathy, diabetic eye disease was reported to be 12% in the African region (though this figure is not specific to the sub-Saharan region); however, by comparison, the reported figure was less than 18% reported for the Eastern Mediterranean region [16]. More detailed and robust prevalence data is needed for those living in sub-Saharan countries as regards diabetes-related eye disease.

Identification of an increase in diabetic retinopathy in the Q3 ancestry may be seen as a future research avenue to identify novel risk alleles for each of those conditions in the drive towards personalized medicine. It has been noted, however, that the Qatari ancestry may differ to that seen in a Caucasian population [6, 14], with studies showing more than 30,000-year divergence between European and Qatari ancestries [17]; therefore, the relevance of any identified novel risk alleles may be restricted to the Middle East. How large a contribution the high level of consanguinity (which is known to exist in the Qatari population) plays is unclear [18]. However, it has been reported that longer runs of homozygosity are found in Qatar, reflecting substantial consanguinity [4] and leading to an increased rate of deleterious variants [19].

Whilst heritability has been estimated to be as high as 27% for diabetic retinopathy (and 52% for the proliferative form) [20, 21], efforts to unravel the genetics using candidate genes, linkage, and GWAS have been unable to identify genes that can be replicated across studies and across ethnic populations [22]. A 2015 literature review looking for associations of gene variants and diabetic retinopathy encompassing diverse ethnic populations found that, whilst a number of the individual studies reviewed reported significant associations between various polymorphisms and diabetic retinopathy, many of the results were conflicting and that no conclusion regarding a clear association with any risk allele could be drawn [23]. The role of SLMAP genetic variants in the susceptibility to diabetes and diabetic retinopathy in the Qatari population has been reported [8]. Despite our conjecture that the association of diabetic retinopathy with the Q3 ancestry may be due to an altered SLMAP allele frequency, no differences in allele frequency were found between the Qatari ancestral groups; however, the retinopathy risk genes in Q3 are not necessarily the same as those in Q1.

With respect to the increased hypertension risk in the Q3 ancestry, it is necessary to determine whether this may require a differing pharmacotherapy approach to the Q1, Q2, and admixed ancestries. However, it is well recognized that the prevalence of hypertension in sub-Saharan populations can be as high as 38% [24].

Hypertension, whilst being a multifactorial disease, has a relatively high heritability [25]. Despite this, a large number of association studies have returned inconsistent results [26], and even GWAS studies have been inconclusive [27, 28]. This is likely because genetic susceptibility to hypertension is polygenic and complex, influenced by environmental factors as well as genes [29].

This study was limited by the small number of subjects that were included, though the findings for the Q3 ancestry were clear-cut. A larger sample with a replication data set would likely have been definitive; however, it is clear from this study that there are differences between the genotypes. It should also be noted that the distribution of participants in the three groups was skewed and as a consequence less powerful nonparametric testing was undertaken that showed differences between the genotypes; however, there were no differences in dyslipidemia or neuropathy, and a larger study would need to be performed to ensure that this was not a false-negative result. A further issue to be considered is that in GWAS studies genetically related individuals should be avoided if the cohort is small because of the risk of bias, noted previously in this population [4]; however, this would have been taken into account in part by the fact that all of the ancestral genotypes were recruited in the same way with their family members. In addition, it has been shown that runs of homozygosity in some individuals reflect substantial consanguinity. However, the variance in runs of homozygosity is exceptionally high, and the degree of identity-by-descent sharing generally appears to be lower than expected for a population in which nearly half of marriages are between first cousins [4].

In conclusion, genotype stratification in this Middle Eastern population identified that the Q3 ancestry genotype arising from sub-Saharan Africa showed an increase in diabetic retinopathy and hypertension. This may represent an avenue for a personalized medicine approach in this population, dictating increased screening or preventative measures.

## Figures and Tables

**Table 1:** Baseline demographic data for the entire cohort of Qatari subjects (*n* = 398), categorized according to the genotype (Q1 Bedouin, Q2 Persian-South Asian, Q3 African, and admixed).

	Q1	Q2	Q3	Admixed	Significance
Gender, *N* (%)					
Male	85 (51.8)	98 (65.8)	12 (38.7)	31 (57.4)	
Female	79 (48.2)	51 (34.2)	19 (61.3)	23 (42.6)	
Age (years), mean (SD)	49.54 (11.05)	49.72 (10.01)	51.80 (9.57)	49.63 (11.23)	0.744
BMI (kg/m^2^), mean (SD)	32.80 (6.37)	31.87 (5.61)	33.40 (7.62)	32.62 (6.24)	0.462
HbA1c (%), median (IQR)	7.1 (2.6)	6.6 (2.9)	7.7 (2.6)	6.7 (3.2)	0.503
Fasting glucose (mmol/l), median (IQR)	6.85 (4.03)	6.05 (3.98)	6.05 (4.40)	5.50 (3.70)	0.100
Diabetes, *N* (%)					
No	65 (39.6)	75 (50.3)	12 (38.7)	26 (48.1)	0.228
Yes	99 (60.4)	74 (49.7)	19 (61.3)	28 (51.9)	0.112

BMI = body mass index; HbA1c = glycated hemoglobin; SD = standard deviation; IQR = interquartile range.

**Table 2:** The relationship of the Qatari genotypes (Q1 Bedouin, Q2 Persian-South Asian, and Q3 African) to diabetes and diabetes complications (microvascular disease) and cardiovascular complications.

T2DM, *N* (%)	Q1 (*N* = 164), 99 (60.4)	Q2, (*N* = 149), 74 (49.7)	Q3, (*N* = 31), 19 (61.3)	Admixed (*N* = 54), 28 (51.9)	*p* value
Gender, *N* (%)					
Male	85 (51.8)	98 (65.8)	12 (38.7)	31 (57.4)	0.014
Female	79 (48.2)	51 (34.2)	19 (61.3)	23 (42.6)	
Diabetes, *N* (%)					
No	65 (39.6)	75 (50.3)	12 (38.7)	26 (48.1)	0.228
Yes	99 (60.4)	74 (49.7)	19 (61.3)	28 (51.9)	
Hypertension, *N* (%)					
No	87 (53.0)	86 (57.7)	9 (29.0)	30 (55.6)	0.035
Yes	77 (47.0)	63 (42.3)	22 (71.0)	24 (44.4)	
Dyslipidemia, *N* (%)					
No	70 (42.7)	66 (44.3)	13 (41.9)	25 (46.3)	0.964
Yes	94 (57.3)	83 (55.7)	18 (58.1)	29 (53.7)	
Diab retinopathy, *N* (%)					
No	139 (84.8)	130 (87.2)	20 (64.5)	43 (79.6)	0.016
Yes	25 (15.2)	19 (12.8)	11 (35.5)	11 (20.4)	
Diab neuropathy, *N* (%)					
No	148 (90.2)	134 (89.9)	26 (83.9)	49 (90.7)	0.724
Yes	16 (9.8)	15 (10.1)	5 (16.1)	5 (9.3)	

**Table 3:** Odds ratio for the risk factors for type 2 diabetes complications.

	Diabetic retinopathy		Diabetic neuropathy	
	OR (95% CI)	*p* value	OR (95% CI)	*p* value
Age	1.07 (1.04-1.09)	<0.001	1.06 (1.03-1.09)	<0.001
Gender-female	1.32 (0.83-2.12)	0.246	0.97 (0.55-1.72)	0.924
BMI (kg/m^2^)	1.04 (0.99-1.07)	0.057	1.00 (0.96-1.05)	0.867
HbA1c (%)	1.55 (1.36-1.77)	<0.001	1.31 (1.14-1.50)	<0.001
Fasting glucose (mmol/l)	1.14 (1.07-1.21)	<0.001	1.15 (1.07-1.23)	<0.001
Hypertension	4.00 (2.38-6.72)	<0.001	4.80 (2.46-9.39)	<0.001
Dyslipidemia	3.11 (1.82-5.32)	<0.001	3.98 (1.95-8.13)	<0.001

**Table 4:** The genetic variation of sarcolemma-associated protein (SLMAP; OMIM ID 602701) in the Q1, Q2, and Q3 ancestries of the Qatari population.

Population	Chromosome	DbSNP	Reference	Alternate	Alternate allele frequency	Depth of coverage
Q1	3	rs17058639	C	T	0.343	222150
Q1	3	rs1057719	A	G	0.357	207986
Q1	3	rs1043045	T	C	0.361	222911
Q2	3	rs17058639	C	T	0.343	222150
Q2	3	rs1057719	A	G	0.357	207986
Q2	3	rs1043045	T	C	0.361	222911
Q3	3	rs17058639	C	T	0.343	222150
Q3	3	rs1057719	A	G	0.357	207986
Q3	3	rs1043045	T	C	0.361	222911

## Data Availability

All data can be made available upon written request to the corresponding author.

## References

[1] Gloyn A. L., Drucker D. J. (2018). Precision medicine in the management of type 2 diabetes. *The Lancet Diabetes & Endocrinology*.

[2] Alhyas L., McKay A., Balasanthiran A., Majeed A. (2011). Prevalences of overweight, obesity, hyperglycaemia, hypertension and dyslipidaemia in the Gulf: systematic review. *JRSM Short Reports*.

[3] Shigidi M. M., Fituri O. M., Chandy S. K., Asim M., Al Malki H. A., Rashed A. H. (2011). Peritoneal dialysis, an expanding mode of renal replacement therapy in Qatar. *Saudi journal of kidney diseases and transplantation : an official publication of the Saudi Center for Organ Transplantation, Saudi Arabia*.

[4] Hunter-Zinck H., Musharoff S., Salit J. (2010). Population genetic structure of the people of Qatar. *American Journal of Human Genetics*.

[5] Rodriguez-Flores J. L., Fakhro K., Hackett N. R. (2014). Exome sequencing identifies potential risk variants for Mendelian disorders at high prevalence in Qatar. *Human Mutation*.

[6] O’Beirne S. L., Salit J., Rodriguez-Flores J. L. (2016). Type 2 diabetes risk allele loci in the Qatari population. *PLoS One*.

[7] O’Beirne S. L., Salit J., Rodriguez-Flores J. L. (2018). Exome sequencing-based identification of novel type 2 diabetes risk allele loci in the Qatari population. *PLoS One*.

[8] Upadhyay R., Robay A., Fakhro K. (2015). Role of SLMAP genetic variants in susceptibility of diabetes and diabetic retinopathy in Qatari population. *Journal of Translational Medicine*.

[9] Sivadas A., Scaria V. (2018). Pharmacogenomic survey of Qatari populations using whole-genome and exome sequences. *The Pharmacogenomics Journal*.

[10] Zayed H. (2016). The Qatar genome project: translation of whole-genome sequencing into clinical practice. *International Journal of Clinical Practice*.

[11] Deckers J. G. M., Schellevis F. G., Fleming D. M. (2010). WHO diagnostic criteria as a validation tool for the diagnosis of diabetes mellitus: a study in five European countries. *European Journal of General Practice*.

[12] Young M. J., Breddy J. L., Veves A., Boulton A. J. M. (1994). The prediction of diabetic neuropathic foot ulceration using vibration perception thresholds. A prospective study. *Diabetes Care*.

[13] Ahlqvist E., Storm P., Käräjämäki A. (2018). Novel subgroups of adult-onset diabetes and their association with outcomes: a data-driven cluster analysis of six variables. *The Lancet Diabetes & Endocrinology*.

[14] Tomei S., Mamtani R., Al Ali R. (2015). Obesity susceptibility loci in Qataris, a highly consanguineous Arabian population. *Journal of Translational Medicine*.

[15] Bener A., Darwish S., Al-Hamaq A. O., Mohammad R. M., Yousafzai M. T. (2013). Association of PPAR*γ*2 gene variant Pro12Ala polymorphism with hypertension and obesity in the aboriginal Qatari population known for being consanguineous. *The Application of Clinical Genetics*.

[16] DF Diabetes Atlas http://www.diabetesatlas.org.

[17] Rodriguez-Flores J. L., Fakhro K., Agosto-Perez F. (2016). Indigenous Arabs are descendants of the earliest split from ancient Eurasian populations. *Genome Research*.

[18] Fahiminiya S., Almuriekhi M., Nawaz Z. (2014). Whole exome sequencing unravels disease-causing genes in consanguineous families in Qatar. *Clinical Genetics*.

[19] Mezzavilla M., Vozzi D., Badii R. (2015). Increased rate of deleterious variants in long runs of homozygosity of an inbred population from Qatar. *Human Heredity*.

[20] Arar N. H., Freedman B. I., Adler S. G. (2008). Heritability of the severity of diabetic retinopathy: the FIND-Eye study. *Investigative Ophthalmology & Visual Science*.

[21] Hietala K., Forsblom C., Summanen P., Groop P. H., on behalf of the FinnDiane Study Group (2008). Heritability of proliferative diabetic retinopathy. *Diabetes*.

[22] Simo-Servat O., Hernandez C., Simo R. (2013). Genetics in diabetic retinopathy: current concepts and new insights. *Current Genomics*.

[23] Schwartz S., Hampton B., Brantley M., Flynn H. (2015). Update on genetics and diabetic retinopathy. *Clinical Ophthalmology*.

[24] Guwatudde D., Nankya-Mutyoba J., Kalyesubula R. (2015). The burden of hypertension in sub-Saharan Africa: a four-country cross sectional study. *BMC Public Health*.

[25] Harrap S. B. (1994). Hypertension: genes versus environment. *Lancet*.

[26] Sõber S., Org E., Kepp K. (2009). Targeting 160 candidate genes for blood pressure regulation with a genome-wide genotyping array. *PLoS One*.

[27] Ehret G. B., Morrison A. C., O'Connor A. A. (2008). Replication of the Wellcome Trust genome-wide association study of essential hypertension: the Family Blood Pressure Program. *European Journal of Human Genetics*.

[28] Levy D., Larson M. G., Benjamin E. J. (2007). Framingham Heart Study 100K Project: genome-wide associations for blood pressure and arterial stiffness. *BMC Med Genet*.

[29] Cowley A. W. (2006). The genetic dissection of essential hypertension. *Nature Reviews Genetics*.

